# 1-[(6-Chloro-3-pyrid­yl)meth­yl]-*N*-(4-ethoxy­phen­yl)-3-phenyl-1*H*-pyrazole-5-carboxamide

**DOI:** 10.1107/S1600536809010290

**Published:** 2009-03-25

**Authors:** Zheng Tang, Xiao-Liang Ding, Wen-Liang Dong, Bao-Xiang Zhao

**Affiliations:** a Submarine College of Navy, Qingdao 266071, People’s Republic of China; b College of Advanced Professional Technology, Qingdao University, Qingdao 266061, People’s Republic of China; c School of Pharmaceutical Sciences, Shandong University of Traditional Chinese Medicine, Jinan 250355, People’s Republic of China; d School of Chemistry and Chemical Engineering, Shandong University, Jinan 250100, People’s Republic of China

## Abstract

In the title compound, C_24_H_21_ClN_4_O_2_, the pyrazole ring makes dihedral angles of 7.70 (11), 89.17 (11) and 40.68 (11)° with the phenyl, pyridine and ethoxy­phenyl rings, respectively. There are some intra­molecular C—H⋯O and C—H⋯π bonds giving rigidity to the mol­ecule, while weak inter­molecular N—H⋯N and C—H⋯π hydrogen bonds link the mol­ecules into a two-dimensional structure.

## Related literature

For the biological properties of pyrazole derivatives, see: Jia *et al.* (2004 ▶); Wei *et al.* (2006 ▶); Xia *et al.* (2007 ▶). For the synthesis and bioactivity evaluation of pyrazole derivatives, see: Zhang *et al.* (2008 ▶); Zhao *et al.* (2008 ▶); Tang *et al.* (2007 ▶). 
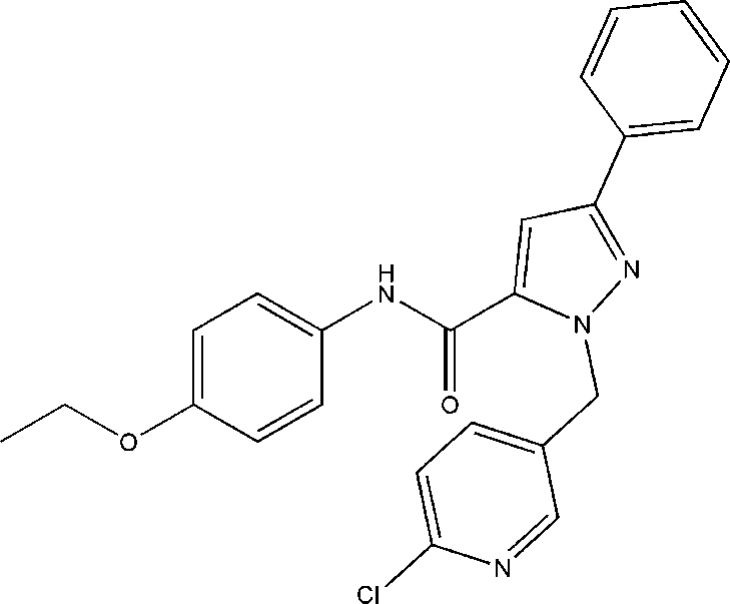


## Experimental

### 

#### Crystal data

C_24_H_21_ClN_4_O_2_
*M* *_r_* = 432.90
Monoclinic, 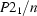

*a* = 10.0697 (12) Å
*b* = 5.1399 (6) Å
*c* = 40.990 (5) Å
β = 96.446 (2)°
*V* = 2108.1 (4) Å^3^
*Z* = 4
Mo *K*α radiation
μ = 0.21 mm^−1^
*T* = 298 K
0.15 × 0.12 × 0.10 mm

#### Data collection

Bruker SMART CCD area-detector diffractometer
Absorption correction: multi-scan (*SADABS*; Bruker, 2005 ▶) *T*_min_ = 0.96, *T*_max_ = 0.98
10472 measured reflections
3699 independent reflections
2571 reflections with *I* > 2σ(*I*)
*R*_int_ = 0.031

#### Refinement

*R*[*F* ^2^ > 2σ(*F* ^2^)] = 0.040
*wR*(*F* ^2^) = 0.104
*S* = 1.03
3699 reflections
280 parameters
H-atom parameters constrained
Δρ_max_ = 0.14 e Å^−3^
Δρ_min_ = −0.23 e Å^−3^

### 

Data collection: *SMART* (Bruker, 2005 ▶); cell refinement: *SAINT* (Bruker, 2005 ▶); data reduction: *SAINT*; program(s) used to solve structure: *SHELXS97* (Sheldrick, 2008 ▶); program(s) used to refine structure: *SHELXL97* (Sheldrick, 2008 ▶); molecular graphics: *XP* in *SHELXTL* (Sheldrick, 2008 ▶); software used to prepare material for publication: *SHELXL97*.

## Supplementary Material

Crystal structure: contains datablocks I, global. DOI: 10.1107/S1600536809010290/bg2246sup1.cif

Structure factors: contains datablocks I. DOI: 10.1107/S1600536809010290/bg2246Isup2.hkl

Additional supplementary materials:  crystallographic information; 3D view; checkCIF report

## Figures and Tables

**Table 1:** Hydrogen-bond geometry (Å, °)

*D*—H⋯*A*	*D*—H	H⋯*A*	*D*⋯*A*	*D*—H⋯*A*
C10—H10*B*⋯O1	0.97	2.35	2.876 (3)	114
C18—H18⋯O1	0.93	2.31	2.861 (3)	118
C12—H12⋯*Cg*1	0.93	2.74	3.354 (2)	125
N4—H4⋯N3^i^	0.86	2.59	3.406 (2)	159
C23—H23*A*⋯*Cg*2^ii^	0.97	2.71	3.571 (3)	149

Symmetry codes: (i) 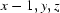; (ii) 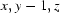. *Cg*1 and *Cg*2 are the centroids of the N1/N2/C1–C3 and C17–C22 rings, respectively.

**Fig. 1.**
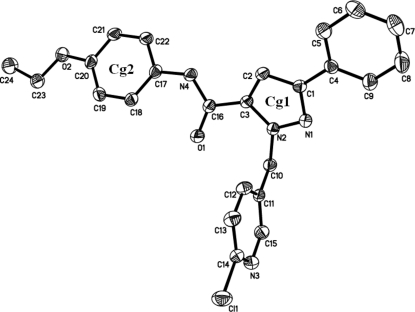
The molecular structure of the title compound, showing atom and centroid labels. Displacement ellipsoids drawn at the 50% probability level for non-H atoms.

## supplementary crystallographic information

### Comment

Pyrazole framework plays an essential role in biologically active compounds. Many pyrazole derivatives are known to exhibit a wide range of biological properties such as anticoagulant (Jia *et al.*, 2004), and antitumour (Wei *et al.*, 2006; Xia *et al.* (2007)) activities. As part of our continuing project of the study on the synthesis and evaluation of pyrazole derivatives (Tang *et al.*, 2007 and Zhao *et al.*,2008; Zhang *et al.*, 2008), we report here the synthesis and crystal structure of the title compound C_24_H_21_ClN_4_O_2_. The pyrazole ring makes dihedral angles of 7.70 (11)°, 89.17 (11)° and 40.68 (11)° with the phenyl, pyridine and ethoxyphenyl rings, respectively. There are some intramolecular C—H···O and C—H···π bonds giving rigidity to the molecule (first three entries in Table 1), while weak intermolecular N—H···N and C—H···π hydrogen bonds (last two entries in Table 1) link the molecules into a 2D structure.

### Experimental

1-(6-Chloropyridin-3-ylmethyl)-3-phenyl-1*H*-pyrazole-5-carboxylic acid (0.31 g, 1 mmol) and thionyl chloride (0.60 g, 5 mmol) were added to a flask with a condenser and heated to reflux for 4 h. After completion of the reaction (by TLC monitoring), the excess thionyl chloride was evaporated under reduced pressure. To the solution of the crude product, 1-((6-chloropyridin-3-yl)methyl)-3-phenyl-1*H*-pyrazole-5-carbonyl chloride (0.332 g, 1 mmol), and triethylamine (0.15 g, 1 mmol) in dichloromethane (20 ml), the solution of 4-ethoxyaniline (0.14 g, 1 mmol) in dichloromethane (20 ml) was added and the mixture was stirred for 20 h at room temperature. Then the mixture was washed with water (20 ml *x* 3). After dried over anhydrous MgSO_4_ the mixture was filtered and the filtrate obtained was concentrated under reduced pressure to obtain the corresponding crude product. The product was purified by column chromatography on silica gel using mixture of dichloromethane and ethyl acetate (10/1) as eluent (yield 43%). Crystals suitable for X-ray diffraction were obtained by slow evaporation of a solution of the solid dissolved in ethyl acetate/hexane at room temperature for 10 days.

### Refinement

All H atoms were placed in calculated positions and refined as riding, with C—H = 0.93–0.97 Å, N—H = 0.86Å and with *U*_iso_(H)=1.2Ueq(C, N) or 1.5Ueq(C) for methyl H atoms.

### Crystal data

**Table:**

C_24_H_21_ClN_4_O_2_	*F*(000) = 904
*M**_r_* = 432.90	*D*_x_ = 1.364 Mg m^−^^3^
Monoclinic, *P*2_1_/*n*	Mo *K*α radiation, λ = 0.71073 Å
Hall symbol: -P 2yn	Cell parameters from 2064 reflections
*a* = 10.0697 (12) Å	θ = 2.7–22.1°
*b* = 5.1399 (6) Å	µ = 0.21 mm^−^^1^
*c* = 40.990 (5) Å	*T* = 298 K
β = 96.446 (2)°	Block, colourless
*V* = 2108.1 (4) Å^3^	0.15 × 0.12 × 0.10 mm
*Z* = 4	

### Data collection

**Table:**

Bruker SMART CCD area-detector diffractometer	3699 independent reflections
Radiation source: fine-focus sealed tube	2571 reflections with *I* > 2σ(*I*)
graphite	*R*_int_ = 0.031
φ and ω scans	θ_max_ = 25.0°, θ_min_ = 1.0°
Absorption correction: multi-scan (*SADABS*; Bruker, 2005)	*h* = −9→11
*T*_min_ = 0.96, *T*_max_ = 0.98	*k* = −6→6
10472 measured reflections	*l* = −46→48

### Refinement

**Table:**

Refinement on *F*^2^	Primary atom site location: structure-invariant direct methods
Least-squares matrix: full	Secondary atom site location: difference Fourier map
*R*[*F*^2^ > 2σ(*F*^2^)] = 0.040	Hydrogen site location: inferred from neighbouring sites
*wR*(*F*^2^) = 0.104	H-atom parameters constrained
*S* = 1.03	*w* = 1/[σ^2^(*F*_o_^2^) + (0.0455*P*)^2^ + 0.3478*P*] where *P* = (*F*_o_^2^ + 2*F*_c_^2^)/3
3699 reflections	(Δ/σ)_max_ < 0.001
280 parameters	Δρ_max_ = 0.14 e Å^−^^3^
0 restraints	Δρ_min_ = −0.22 e Å^−^^3^

### Special details

**Table:**

Geometry. All e.s.d.'s (except the e.s.d. in the dihedral angle between two l.s. planes) are estimated using the full covariance matrix. The cell e.s.d.'s are taken into account individually in the estimation of e.s.d.'s in distances, angles and torsion angles; correlations between e.s.d.'s in cell parameters are only used when they are defined by crystal symmetry. An approximate (isotropic) treatment of cell e.s.d.'s is used for estimating e.s.d.'s involving l.s. planes.
Refinement. Refinement of *F*^2^ against ALL reflections. The weighted *R*-factor *wR* and goodness of fit *S* are based on *F*^2^, conventional *R*-factors *R* are based on *F*, with *F* set to zero for negative *F*^2^. The threshold expression of *F*^2^ > σ(*F*^2^) is used only for calculating *R*-factors(gt) *etc*. and is not relevant to the choice of reflections for refinement. *R*-factors based on *F*^2^ are statistically about twice as large as those based on *F*, and *R*- factors based on ALL data will be even larger.

### Fractional atomic coordinates and isotropic or equivalent isotropic displacement parameters (Å^2^)

**Table:**

	*x*	*y*	*z*	*U*_iso_*/*U*_eq_	
Cl1	0.71782 (6)	−0.22109 (13)	0.164051 (17)	0.0697 (2)	
O1	0.10720 (14)	0.0159 (3)	0.08714 (4)	0.0596 (4)	
O2	−0.32793 (14)	−0.7133 (3)	0.00202 (4)	0.0592 (4)	
N1	0.15163 (15)	0.5760 (3)	0.16155 (4)	0.0401 (4)	
N2	0.14531 (15)	0.4370 (3)	0.13344 (4)	0.0382 (4)	
N3	0.60325 (16)	0.1539 (4)	0.12864 (4)	0.0478 (5)	
N4	−0.10977 (15)	−0.0112 (3)	0.09696 (4)	0.0453 (4)	
H4	−0.1670	0.0486	0.1091	0.054*	
C1	0.04487 (18)	0.5012 (4)	0.17605 (5)	0.0384 (5)	
C2	−0.02871 (19)	0.3133 (4)	0.15696 (5)	0.0420 (5)	
H2	−0.1070	0.2322	0.1616	0.050*	
C3	0.03779 (18)	0.2730 (4)	0.13003 (5)	0.0381 (5)	
C4	0.02032 (19)	0.6110 (4)	0.20807 (5)	0.0395 (5)	
C5	−0.0794 (2)	0.5097 (4)	0.22505 (5)	0.0509 (6)	
H5	−0.1304	0.3704	0.2162	0.061*	
C6	−0.1040 (2)	0.6129 (5)	0.25494 (6)	0.0621 (7)	
H6	−0.1718	0.5436	0.2659	0.075*	
C7	−0.0295 (3)	0.8158 (5)	0.26846 (6)	0.0660 (7)	
H7	−0.0462	0.8846	0.2886	0.079*	
C8	0.0708 (3)	0.9179 (5)	0.25205 (6)	0.0695 (7)	
H8	0.1224	1.0551	0.2612	0.083*	
C9	0.0951 (2)	0.8179 (4)	0.22210 (5)	0.0545 (6)	
H9	0.1624	0.8897	0.2111	0.065*	
C10	0.25901 (18)	0.4539 (4)	0.11441 (5)	0.0409 (5)	
H10A	0.2898	0.6327	0.1143	0.049*	
H10B	0.2309	0.4033	0.0919	0.049*	
C11	0.37272 (18)	0.2810 (4)	0.12835 (4)	0.0354 (5)	
C12	0.3565 (2)	0.0878 (4)	0.15098 (5)	0.0452 (5)	
H12	0.2738	0.0643	0.1586	0.054*	
C13	0.4616 (2)	−0.0693 (4)	0.16222 (5)	0.0499 (6)	
H13	0.4518	−0.2001	0.1774	0.060*	
C14	0.58170 (19)	−0.0273 (4)	0.15031 (5)	0.0435 (5)	
C15	0.4985 (2)	0.3043 (4)	0.11817 (5)	0.0457 (5)	
H15	0.5112	0.4336	0.1030	0.055*	
C16	0.01583 (19)	0.0825 (4)	0.10273 (5)	0.0413 (5)	
C17	−0.15751 (19)	−0.1981 (4)	0.07291 (5)	0.0417 (5)	
C18	−0.0756 (2)	−0.3663 (4)	0.05794 (5)	0.0464 (5)	
H18	0.0164	−0.3612	0.0637	0.056*	
C19	−0.1289 (2)	−0.5423 (4)	0.03440 (5)	0.0486 (5)	
H19	−0.0725	−0.6532	0.0245	0.058*	
C20	−0.2651 (2)	−0.5540 (4)	0.02558 (5)	0.0462 (5)	
C21	−0.3474 (2)	−0.3877 (5)	0.04087 (5)	0.0540 (6)	
H21	−0.4395	−0.3950	0.0353	0.065*	
C22	−0.2944 (2)	−0.2126 (4)	0.06405 (5)	0.0521 (6)	
H22	−0.3511	−0.1021	0.0740	0.062*	
C23	−0.2494 (2)	−0.8951 (5)	−0.01383 (6)	0.0588 (6)	
H23A	−0.2020	−1.0104	0.0022	0.071*	
H23B	−0.1844	−0.8044	−0.0254	0.071*	
C24	−0.3440 (3)	−1.0481 (6)	−0.03759 (6)	0.0780 (8)	
H24A	−0.4154	−1.1156	−0.0264	0.117*	
H24B	−0.2969	−1.1896	−0.0463	0.117*	
H24C	−0.3802	−0.9367	−0.0552	0.117*	

### Atomic displacement parameters (Å^2^)

**Table:**

	*U*^11^	*U*^22^	*U*^33^	*U*^12^	*U*^13^	*U*^23^
Cl1	0.0448 (3)	0.0661 (4)	0.0967 (5)	0.0083 (3)	0.0017 (3)	0.0136 (4)
O1	0.0435 (9)	0.0673 (11)	0.0702 (11)	−0.0103 (8)	0.0162 (8)	−0.0280 (9)
O2	0.0513 (9)	0.0624 (10)	0.0624 (10)	−0.0076 (8)	0.0004 (7)	−0.0262 (8)
N1	0.0388 (9)	0.0392 (10)	0.0424 (10)	−0.0022 (8)	0.0045 (7)	−0.0058 (8)
N2	0.0367 (9)	0.0396 (10)	0.0387 (9)	−0.0042 (8)	0.0063 (7)	−0.0059 (8)
N3	0.0407 (10)	0.0492 (11)	0.0549 (11)	−0.0024 (9)	0.0119 (8)	−0.0025 (9)
N4	0.0378 (9)	0.0557 (11)	0.0430 (10)	−0.0096 (8)	0.0068 (7)	−0.0140 (9)
C1	0.0320 (10)	0.0398 (12)	0.0431 (12)	0.0012 (9)	0.0028 (9)	−0.0005 (10)
C2	0.0333 (11)	0.0481 (13)	0.0446 (12)	−0.0057 (10)	0.0043 (9)	−0.0059 (10)
C3	0.0320 (10)	0.0415 (12)	0.0403 (11)	−0.0030 (9)	0.0013 (9)	−0.0033 (9)
C4	0.0369 (11)	0.0400 (12)	0.0408 (11)	0.0063 (9)	0.0011 (9)	−0.0026 (9)
C5	0.0520 (13)	0.0550 (14)	0.0461 (13)	−0.0029 (11)	0.0076 (10)	−0.0045 (11)
C6	0.0677 (16)	0.0717 (17)	0.0502 (14)	0.0057 (14)	0.0210 (12)	0.0006 (13)
C7	0.0872 (19)	0.0653 (17)	0.0466 (14)	0.0106 (15)	0.0123 (13)	−0.0111 (13)
C8	0.0876 (19)	0.0611 (17)	0.0595 (16)	−0.0088 (15)	0.0065 (14)	−0.0214 (13)
C9	0.0589 (14)	0.0545 (15)	0.0510 (14)	−0.0081 (12)	0.0104 (11)	−0.0106 (12)
C10	0.0406 (11)	0.0427 (12)	0.0406 (12)	−0.0054 (10)	0.0101 (9)	−0.0014 (10)
C11	0.0357 (11)	0.0351 (11)	0.0359 (11)	−0.0049 (9)	0.0057 (8)	−0.0059 (9)
C12	0.0369 (12)	0.0518 (14)	0.0479 (12)	−0.0053 (10)	0.0095 (9)	0.0053 (11)
C13	0.0469 (13)	0.0509 (14)	0.0517 (13)	−0.0037 (11)	0.0048 (10)	0.0130 (11)
C14	0.0351 (11)	0.0435 (13)	0.0508 (13)	−0.0016 (9)	0.0006 (9)	−0.0057 (11)
C15	0.0461 (13)	0.0454 (13)	0.0471 (13)	−0.0039 (10)	0.0129 (10)	0.0043 (10)
C16	0.0391 (12)	0.0418 (12)	0.0427 (12)	−0.0047 (10)	0.0032 (9)	−0.0041 (10)
C17	0.0436 (12)	0.0447 (13)	0.0366 (11)	−0.0090 (10)	0.0032 (9)	−0.0046 (10)
C18	0.0388 (12)	0.0456 (13)	0.0528 (13)	−0.0020 (10)	−0.0040 (10)	−0.0053 (11)
C19	0.0488 (13)	0.0446 (13)	0.0520 (13)	0.0016 (11)	0.0034 (10)	−0.0096 (11)
C20	0.0485 (13)	0.0457 (13)	0.0437 (12)	−0.0117 (11)	0.0019 (10)	−0.0092 (10)
C21	0.0366 (12)	0.0693 (16)	0.0562 (14)	−0.0104 (11)	0.0048 (10)	−0.0195 (12)
C22	0.0412 (12)	0.0626 (15)	0.0536 (14)	−0.0070 (11)	0.0105 (10)	−0.0195 (12)
C23	0.0591 (14)	0.0627 (16)	0.0562 (14)	−0.0098 (13)	0.0135 (11)	−0.0187 (12)
C24	0.0737 (17)	0.092 (2)	0.0695 (17)	−0.0161 (16)	0.0130 (13)	−0.0413 (16)

### Geometric parameters (Å, °)

**Table:**

Cl1—C14	1.736 (2)	C9—H9	0.9300
O1—C16	1.226 (2)	C10—C11	1.510 (3)
O2—C20	1.366 (2)	C10—H10A	0.9700
O2—C23	1.427 (2)	C10—H10B	0.9700
N1—C1	1.342 (2)	C11—C12	1.381 (3)
N1—N2	1.351 (2)	C11—C15	1.382 (3)
N2—C3	1.367 (2)	C12—C13	1.369 (3)
N2—C10	1.458 (2)	C12—H12	0.9300
N3—C14	1.322 (3)	C13—C14	1.371 (3)
N3—C15	1.339 (3)	C13—H13	0.9300
N4—C16	1.349 (2)	C15—H15	0.9300
N4—C17	1.421 (2)	C17—C18	1.385 (3)
N4—H4	0.8600	C17—C22	1.388 (3)
C1—C2	1.401 (3)	C18—C19	1.386 (3)
C1—C4	1.475 (3)	C18—H18	0.9300
C2—C3	1.370 (3)	C19—C20	1.379 (3)
C2—H2	0.9300	C19—H19	0.9300
C3—C16	1.485 (3)	C20—C21	1.388 (3)
C4—C5	1.386 (3)	C21—C22	1.372 (3)
C4—C9	1.390 (3)	C21—H21	0.9300
C5—C6	1.383 (3)	C22—H22	0.9300
C5—H5	0.9300	C23—C24	1.506 (3)
C6—C7	1.365 (3)	C23—H23A	0.9700
C6—H6	0.9300	C23—H23B	0.9700
C7—C8	1.379 (3)	C24—H24A	0.9600
C7—H7	0.9300	C24—H24B	0.9600
C8—C9	1.378 (3)	C24—H24C	0.9600
C8—H8	0.9300		
			
C20—O2—C23	118.55 (17)	C13—C12—C11	120.33 (18)
C1—N1—N2	105.24 (15)	C13—C12—H12	119.8
N1—N2—C3	111.98 (14)	C11—C12—H12	119.8
N1—N2—C10	117.37 (15)	C12—C13—C14	117.8 (2)
C3—N2—C10	129.97 (16)	C12—C13—H13	121.1
C14—N3—C15	116.06 (17)	C14—C13—H13	121.1
C16—N4—C17	126.88 (17)	N3—C14—C13	124.61 (19)
C16—N4—H4	116.6	N3—C14—Cl1	116.05 (15)
C17—N4—H4	116.6	C13—C14—Cl1	119.34 (17)
N1—C1—C2	110.48 (17)	N3—C15—C11	124.67 (19)
N1—C1—C4	120.47 (17)	N3—C15—H15	117.7
C2—C1—C4	129.03 (17)	C11—C15—H15	117.7
C3—C2—C1	106.18 (17)	O1—C16—N4	123.54 (18)
C3—C2—H2	126.9	O1—C16—C3	121.32 (17)
C1—C2—H2	126.9	N4—C16—C3	115.13 (17)
N2—C3—C2	106.11 (16)	C18—C17—C22	118.31 (19)
N2—C3—C16	122.22 (16)	C18—C17—N4	123.88 (18)
C2—C3—C16	131.48 (17)	C22—C17—N4	117.81 (18)
C5—C4—C9	117.98 (19)	C17—C18—C19	120.80 (19)
C5—C4—C1	120.42 (18)	C17—C18—H18	119.6
C9—C4—C1	121.61 (18)	C19—C18—H18	119.6
C6—C5—C4	120.8 (2)	C20—C19—C18	120.46 (19)
C6—C5—H5	119.6	C20—C19—H19	119.8
C4—C5—H5	119.6	C18—C19—H19	119.8
C7—C6—C5	120.5 (2)	O2—C20—C19	125.40 (19)
C7—C6—H6	119.7	O2—C20—C21	115.80 (18)
C5—C6—H6	119.7	C19—C20—C21	118.78 (19)
C6—C7—C8	119.5 (2)	C22—C21—C20	120.72 (19)
C6—C7—H7	120.3	C22—C21—H21	119.6
C8—C7—H7	120.3	C20—C21—H21	119.6
C9—C8—C7	120.4 (2)	C21—C22—C17	120.9 (2)
C9—C8—H8	119.8	C21—C22—H22	119.5
C7—C8—H8	119.8	C17—C22—H22	119.5
C8—C9—C4	120.8 (2)	O2—C23—C24	107.08 (19)
C8—C9—H9	119.6	O2—C23—H23A	110.3
C4—C9—H9	119.6	C24—C23—H23A	110.3
N2—C10—C11	111.72 (16)	O2—C23—H23B	110.3
N2—C10—H10A	109.3	C24—C23—H23B	110.3
C11—C10—H10A	109.3	H23A—C23—H23B	108.6
N2—C10—H10B	109.3	C23—C24—H24A	109.5
C11—C10—H10B	109.3	C23—C24—H24B	109.5
H10A—C10—H10B	107.9	H24A—C24—H24B	109.5
C12—C11—C15	116.50 (18)	C23—C24—H24C	109.5
C12—C11—C10	122.38 (16)	H24A—C24—H24C	109.5
C15—C11—C10	121.09 (18)	H24B—C24—H24C	109.5
			
C1—N1—N2—C3	−0.8 (2)	C11—C12—C13—C14	0.1 (3)
C1—N1—N2—C10	−172.37 (16)	C15—N3—C14—C13	0.2 (3)
N2—N1—C1—C2	0.2 (2)	C15—N3—C14—Cl1	−179.67 (15)
N2—N1—C1—C4	178.97 (16)	C12—C13—C14—N3	−0.2 (3)
N1—C1—C2—C3	0.5 (2)	C12—C13—C14—Cl1	179.70 (16)
C4—C1—C2—C3	−178.14 (19)	C14—N3—C15—C11	−0.2 (3)
N1—N2—C3—C2	1.2 (2)	C12—C11—C15—N3	0.1 (3)
C10—N2—C3—C2	171.32 (18)	C10—C11—C15—N3	−178.05 (18)
N1—N2—C3—C16	−174.30 (17)	C17—N4—C16—O1	0.5 (3)
C10—N2—C3—C16	−4.1 (3)	C17—N4—C16—C3	−178.72 (18)
C1—C2—C3—N2	−1.0 (2)	N2—C3—C16—O1	18.9 (3)
C1—C2—C3—C16	173.9 (2)	C2—C3—C16—O1	−155.2 (2)
N1—C1—C4—C5	−171.48 (18)	N2—C3—C16—N4	−161.82 (18)
C2—C1—C4—C5	7.0 (3)	C2—C3—C16—N4	24.0 (3)
N1—C1—C4—C9	8.7 (3)	C16—N4—C17—C18	19.0 (3)
C2—C1—C4—C9	−172.8 (2)	C16—N4—C17—C22	−161.1 (2)
C9—C4—C5—C6	0.4 (3)	C22—C17—C18—C19	0.8 (3)
C1—C4—C5—C6	−179.4 (2)	N4—C17—C18—C19	−179.33 (19)
C4—C5—C6—C7	−0.6 (4)	C17—C18—C19—C20	−0.4 (3)
C5—C6—C7—C8	0.1 (4)	C23—O2—C20—C19	3.9 (3)
C6—C7—C8—C9	0.6 (4)	C23—O2—C20—C21	−177.9 (2)
C7—C8—C9—C4	−0.7 (4)	C18—C19—C20—O2	177.8 (2)
C5—C4—C9—C8	0.2 (3)	C18—C19—C20—C21	−0.4 (3)
C1—C4—C9—C8	−180.0 (2)	O2—C20—C21—C22	−177.6 (2)
N1—N2—C10—C11	80.0 (2)	C19—C20—C21—C22	0.8 (3)
C3—N2—C10—C11	−89.7 (2)	C20—C21—C22—C17	−0.3 (4)
N2—C10—C11—C12	14.6 (3)	C18—C17—C22—C21	−0.5 (3)
N2—C10—C11—C15	−167.36 (17)	N4—C17—C22—C21	179.7 (2)
C15—C11—C12—C13	0.0 (3)	C20—O2—C23—C24	178.38 (19)
C10—C11—C12—C13	178.07 (19)		

### Hydrogen-bond geometry (Å, °)

**Table:**

*D*—H···*A*	*D*—H	H···*A*	*D*···*A*	*D*—H···*A*
C10—H10B···O1	0.97	2.35	2.876 (3)	114
C18—H18···O1	0.93	2.31	2.861 (3)	118
C12—H12···Cg1	0.93	2.74	3.354 (2)	125
N4—H4···N3^i^	0.86	2.59	3.406 (2)	159
C23—H23A···Cg2^ii^	0.97	2.71	3.571 (3)	149

Symmetry codes: (i) *x*−1, *y*, *z*; (ii) *x*, *y*−1, *z*.
